# Hop Leaves as an Alternative Source of Health-Active Compounds: Effect of Genotype and Drying Conditions

**DOI:** 10.3390/plants11010099

**Published:** 2021-12-29

**Authors:** Valentina Macchioni, Valentina Picchi, Katya Carbone

**Affiliations:** 1 CREA-Research Centre for Olive, Fruit and Citrus Crops, Via di Fioranello 52, 00134 Rome, Italy; valentina.macchioni14@gmail.com; 2 CREA-Research Centre for Engineering and Agro-Food Processing, Via G. Venezian 26, 20133 Milan, Italy; valentina.picchi@crea.gov.it

**Keywords:** *Humulus lupulus* L., circular economy, drying methods, bioactive compounds

## Abstract

In hop cultivation, one-third of the crop is a valuable product (hop cones), and two-thirds is unexploited biomass, consisting mainly of leaves and stems, which, in a circular economy approach, can be recovered and, once stabilized, supplied to industrial sectors, such as cosmetics, pharmaceuticals and phytotherapy, with high added value. In this regard, this study aimed to investigate the effects of two different drying methods: oven drying (OD) at 45 °C and freeze-drying (FD), on the overall nutraceutical profile (i.e., total phenols, total flavans and total thiols), pigment content (i.e., carotenoids and chlorophylls) and the antioxidant potential of leaves from five different *Humulus lupulus* varieties grown in central Italy. Moreover, attenuated total reflectance infrared (ATR-FTIR) spectroscopy was applied to dried leaf powders to study the influence of both the variety and treatment on their molecular fingerprints. The spectral data were then analyzed by principal component analysis (PCA), which was able to group the samples mainly based on the applied treatment. Considering the overall phytochemical profile, FD appeared to be the most suitable drying method, while OD provided higher carotenoid retention, depending on the genotype considered. Finally, unsupervised chemometric tools (i.e., PCA and hierarchical clustering) revealed that the two main clusters contained subclusters based on the drying treatment applied; these subgroups were related to the susceptibility of the variety to the drying conditions studied.

## 1. Introduction

Hop plants (*Humulus lupulus* L.) are grown almost exclusively for the brewing industry, in which resins and essential oils from female cones are used for aroma [1]. However, the plant has been well-known for its beneficial properties for human health since ancient times. This is due to the plethora of its bioactive compounds (bitter acids, prenyl chalcones, polyphenols, etc.), which are mainly located in the female inflorescences, named hop cones [2]. Consequently, in recent years, hop applications have been increasingly moving beyond beer production, especially within the food sector [3,4,5]. According to the latest FAO estimates, the global area devoted to hop cultivation was around 65,500 ha in 2019, with a production that exceeded 130,000 tons [6]. The European continent contributed decisively to this production, with a volume of almost 68,000 tons, representing 52% of the world hop production [7]. Hop is a perennial climbing plant able to reach seven meters in height. At harvest, one-thirds is valuable product (hop cones) and two-thirds is leftover biomass, consisting primarily of leaves, stems and unremoved hop cones. While hop waste from brewing, named spent hop, has been studied in-depth in recent years [8,9], little attention has been paid to the postharvest hop biomass, which accounts for about 10–15 t/ha annually (2.6 kg/plant) [10] and can be considered a valuable source of functional molecules and nutrients that are still underexploited [11]. In this regard, Rutto et al. [12] demonstrated the potential value of hop biomass beyond the cones (i.e., leaves, bines and unrecovered cones) as promising forage to improve feed efficiency in ruminants. In addition, Afonso et al. [13] underlined the nitrogen richness of hop leaves, making it a good starting material for composting. The authors also provided some suggestions to improve the composting of hop leaves, such as mixing them with other materials (i.e., cow manure and wheat straw; [14]). Recently, Iglesias et al. [15] suggested the use of alcoholic extracts of hop leaves to produce an eco-friendly bee pesticide and underlined the influence of genotype on their secondary metabolite content. These biological activities are related to the bioactive compounds present in hop leaves, such as polyphenols [16]. Muzykiewicz et al. [2] reported a total polyphenol content of hop leaf alcoholic extracts ranging from 0.09 to 6.22 mg GA g^−1^ raw material, depending on the type of solvent used and harvest year considered. Recently, Morcol et al. [17] reported the presence of several bioactive compounds (i.e., bitter acids, prenylcalcones and phenolic acids) in hydroalcoholic extracts of hop leaves from different commercial varieties, highlighting the influence of genotype on their metabolic profile. According to the “Polyphenols Market Size, Share & Trends Analysis Report” (https://www.grandviewresearch.com; accessed: 11 August 2021), “…*the global polyphenols market size was valued at USD 1.28 billion in 2018 and is expected to register an estimated CAGR of 7.2% from 2019 to 2025. In this regard, the Generally Recognized as Safe (GRAS) status awarded to the use of polyphenols in the food and beverage sector is expected to broaden their application scope, from beyond the niche nutraceutical sector to the largescale food and beverage industry*…”. In addition, the food industry has developed increasingly interest for the use of aromatic herbs in cooking or in the formulation of herbal teas [18]. The herbal tea market was valued at USD 3289.67 million in 2020 and is expected to reach USD 4877.80 million by 2028. Recently, Dziedzinski et al. [16] showed the possibility of obtaining functional teas using hop leaves and low concentrations of hop cones to provide a product with a pleasant taste and pro-health properties, and they also emphasized the role of genotype in determining these functional properties. 

In light of these considerations, it seems clear that, through a green technological approach, the exploitation of hop biomass as a source of polyphenols and other valuable molecules, not only for the food sector, can offer the farmer a way to expand the portfolio of products, diversifying business income through the adoption of a virtuous and sustainable management model. However, to industrially exploit the biological properties of this waste as antioxidants, antimicrobials, etc., in food, cosmetic and pharmaceutical formulations, it is necessary to stabilize the fresh plant biomass to avoid degradation processes by eliminating the residual moisture, mainly to diminish microbiological spoilage and increase its shelf life, but also to reduce packaging costs and shipping weights [19]. However, water removal is not the only consequence of most drying operations. During the drying process, in fact, other important changes can occur in the plant matrix related to its qualitative aspects, such as the possible loss or alteration of biologically active compounds, the outcome of which is closely related to the conditions of the process [20,21]. Currently, several drying techniques are available, which affect the quality of the plant matrix in different ways. Convective drying, also conventionally referred to as oven drying (OD), is one of the simplest and cheapest drying methods for stabilizing food matrices with limited rehydration, and at the same time, it is an easy process to scale up [22]. However, this technique has some negative aspects, such as a relatively long duration and high temperature, which, in some cases, can compromise the quality of the final product [23]. On the other hand, freeze-drying (FD) is considered one of the best dehydration techniques for preserving the qualities of the final product, although the process is longer and more expensive than OD [24,25]. In this regard, Roshanak et al. [26] evaluated the effect of different drying methods on the preservation of bioactive compounds in *Camellia sinensis* leaves. Their results showed that OD could preserve more of the antioxidant properties of the plant matrix, while FD provided a better extraction yield of chlorophyll and vitamin C. Once stabilized, for proper exploitation of the plant matrix under study, it is necessary to analyze possible routes based on green technologies for its full use, for example, in the field of functional extracts. In this regard, unconventional and green extraction techniques, such as ultrasound-assisted extraction (UAE) coupled with food-grade solvents, have been successfully applied to recover bioactive compounds from plants to achieve acceptable results in terms of both the yield and environmental sustainability of the process applied [27]. UAE is an innovative nonthermal extraction process that improves solid/liquid mass transfer through acoustic cavitation induced in the liquid medium, thereby increasing extraction efficiency. Other advantages of using this technique are its simplicity, safety, versatility, rapidity and environmental friendliness due to reduced time, energy consumption and solvents [28,29]. As far as we know, there are no literature data on the effect of different drying methods, as well as different genotypes, on the phytochemical profile and antioxidant potential of hop leaves, nor are there any studies on the formulation of functional green extracts from these wastes.

In light of these considerations, the aim of the present study was to evaluate the antioxidant potential and nutraceutical profile of hop leaves from different hop varieties grown in central Italy for the possible recovery and valorization of this waste. To this end, the effects of two biomass stabilization techniques on these parameters were evaluated. Two different drying processes were chosen based on the following rationale. As for the OD technique, it was decided to make the best use of the dryers already present in hop farms. In fact, to better preserve hop cones, both in microbiological and aromatic terms, they were dried directly at the farm by using conventional dryers, operating between 45 and 55 °C, to dry the material quickly at a residual humidity between 8 and 10% The exploitation of these dryers would therefore make it possible to amortize the available equipment without having to foresee additional costs for the purchase of new machinery. On the other hand, FD, as mentioned above, is the technique of choice for the drying of vegetable matrices, as it can better preserve their nutritional components. However, the introduction of this technique to the farm would require an important investment in addition to, among other factors pointed out in the introduction, longer times required by the freeze-drying process. 

For a full understanding of the effects of the drying technique and genotype on the overall metabolic profile of the samples under study, for the first time to our knowledge, Fourier-transform infrared (FTIR) spectroscopy was applied. Ultrasound-assisted ethanolic extracts from hop leaves were also prepared and analyzed for their contents of total phenols, total flavans, total thiols and total pigments (i.e., chlorophylls and carotenoids). Finally, we evaluated the influence of the drying method and genotype, as well as their interactions, on the FTIR profiles, overall nutraceutical content (i.e., total phenols, total flavans, total thiols and total pigments) and antioxidant potential of samples investigated. The interrelationships between the parameters analyzed, the genotype and the drying treatment applied, as well as the relationships among variables, were investigated by unsupervised chemometric tools such as hierarchical cluster analysis (HCA) and principal component analysis (PCA), aiming to establish a guideline for the best recovery of these waste materials and to enhance them through the formulation of final extracts that could be applied in the health industry and beyond.

## 2. Materials and Methods

### 2.1. Chemicals

All reagents used were of analytical spectrophotometric grade (Carlo Erba, Rome, Italy). Folin–Ciocâlteau reagent, 2,2-diphenyl-1-picrylhydrazyl radical (DPPH^•^), vanillin, 2,20-azinobis-(3-ethylbenzothiazolin-6-sulfonic acid) (ABTS^•+^), potassium persulfate and ethanol were purchased from Sigma-Aldrich (Milan, Italy). All other solvents and reagents used were analytically pure. Prior to analysis, all samples were filtered through membrane filters (cellulose acetate) with a pore size of 0.45 µm purchased from Pall (Pall Corporation, Ann Arbor, MI, USA).

### 2.2. Plant Material

*Humulus lupulus* leaves from five hop genotypes (Table 1) grown under organic farming conditions were collected at harvest at the farm I Vizi del Luppolo (Cori, Italy; 41°63′46″ N-12°87′18″ E) and cold-transported to the Food Chemistry and Biotechnology laboratory at CREA Research Centre for Olive, Fruit and Citrus Crops (Rome, Italy). An aliquot of leaves (300 g) of each genotype was subjected to oven drying (OD) at 45 °C (air velocity: 0.6 ms^−1^, relative humidity < 0.5%, system power: 1.4 kW/h; model 600, Memmert GmbH + Co.KG, Schwabach, Germany). This type of drying and the temperature were chosen considering the possibility of exploiting the drying systems for hop cones that are generally present in these farms. The remaining part (300 g) was freeze-dried at −54 °C and 0.075 mbar (model Modulyo 4 K, Edwards, UK). Sample dehydration using all of the methods mentioned above was continued until about 8–10% final moisture content was reached. At the end of each drying treatment, samples were finely milled (sieve 0.5 mm), stored under vacuum and kept protected from light and moisture until analysis. Four replicates for each treatment were carried out.

### 2.3. Fourier-Transform Infrared Spectroscopy (FTIR) Analysis in Attenuated Total Reflectance (ATR) Mode

ATR-FTIR spectra of hop leaves were collected using an iS 50 Nicolet FTIR spectrometer (Thermo Fisher Scientific Inc., Waltham, MA, USA) operating in reflection mode, as described by Macchioni et al. [30] without modifications. IR spectra (wavenumbers ranging from 4000 to 600 cm^−1^) were collected at room temperature, and the background was measured before each sample acquisition. The raw spectra were then processed with the OMNIC^TM^ software (Thermo Fisher Scientific Inc., USA).

### 2.4. Extraction of Bioactive Compounds

Powdered leaf samples were subjected to ultrasound-assisted extraction (UAE) performed in a temperature-controlled sonication bath (UTA-200, Falc, Italy) operating at 40 kHz, according to Carbone et al. [29]. Briefly, samples (1.0 g) were first mixed with 15 mL of ethanol (96%) in test tubes with screw caps on a mechanical shaker (760 rpm; shaking incubator mod. SKI 4; Argolab, Milan, Italy) in the dark and at room temperature (25 °C). Then, the mixture underwent UAE for 30 min at 25 °C under ultrasound irradiation. The resulting extracts were then centrifuged at 6792× *g* for 15 min at 4 °C. Pellets were extracted once again in the same manner. Then, the supernatants were collected and immediately analyzed. For total thiol content (Thl) extraction, 30 mg of dried sample was added to 1.5 mL of 6% metaphosphoric acid. The homogenate was vortexed for 30 s, centrifuged at 12,000 rpm for 15 min at 4 °C and filtered through a 0.45 µm filter.

### 2.5. Analysis of Bioactive Compounds in Hop Leaves

The total polyphenol content (TPC) of the samples analyzed was determined according to Carbone et al. [31], without modifications. The calibration curve was generated with standard solutions of gallic acid in the range 0–100 ppm, and the measures were carried out at 765 nm using a UV–vis spectrophotometer (model 6300 PC, VWR, Milan, Italy). All analyses were performed in triplicate. TPC was expressed as milligrams of gallic acid equivalents per gram of dried sample (mg GAE g^−1^). 

Total flavan content (FLC) was determined following the vanillin assay method, as reported by Carbone et al. [31], without modifications. FLC was calculated from a calibration curve in the range 0–100 ppm, using catechin as a standard. Results were expressed as milligrams of catechin equivalents per gram of dried sample (mg CTE g^−1^). All determinations were performed in triplicate.

The pigment content (i.e., total chlorophyll content, chlorophyll *a* (Chl *a*) and *b* (Chl *b*) and total carotenoids (TC)) of the samples analyzed were determined according to Lichtenthaler and Buschmann [32]. Results were given in μg g^−1^ of dry product. Thl was measured as described by Picchi et al. [33], without modifications. Quantification was performed using a calibration curve of standard solutions of glutathione (GSH) in the range of 0–60 ppm, and results were expressed as mg GSH equivalent per 100 g of dry weight. All analyses were performed in triplicate.

### 2.6. Determination of the Antiradical Capacity (AC)

The radical scavenging power of the samples analyzed was assessed by measuring their ability to scavenge synthetic radicals (e.g., DPPH^•^ and ABTS^•+^). The ABTS radical cation decolorization assay and DPPH^•^ quenching capacity of extracts were determined spectrophotometrically, as reported by Carbone et al. [29], without modifications. Results were expressed as μg mL^−1^ of the extract required to obtain 50% radical scavenging (EC_50_). All determinations were performed in triplicate.

### 2.7. Statistical Analysis

Statistical analysis was performed with SPSS 25.0 software (SPSS, Inc., Chicago, Illinois) and Matlab R2020a software (MathWorks, Inc., Natick, MA, USA). Data were reported as mean ± standard deviation (SD) of four independent experiments with three replicates. Data related to bioactive compounds and antioxidant potential were subjected to analysis of variance (ANOVA) using a factorial model with variety and treatment (V and T, respectively) and V × T interactions. Correlations among all parameters in the dataset were analyzed using Pearson’s correlations (*r*; *p* < 0.05 and *p* < 0.01). Finally, principal component analysis (PCA) and hierarchical cluster analysis (HCA) were used as exploratory chemometric methods to study the data structure and to investigate similarities and hidden patterns among the bioactive compounds in the samples analyzed. Both methods were applied to normalized data (z-scores) to systematically retrieve all chemically relevant information. Clusters were computed by Ward’s method based on Euclidean distance, and ANOVA was used to compare them. Significant differences among clusters were investigated by Tukey’s HSD post hoc test. Significance tests on the variables used to create the clusters indicated whether and how clusters differ for each clustering variable. PCA was used to establish the relationships among all variables under study and to discriminate between different treatments and varieties. It was performed using a data correlation matrix and Varimax rotation between the samples.

PCA was also applied to the preprocessed (smoothing and normalization) ATR-FTIR spectra in the range 4000–800 cm^−1^ to identify spectral regions that allowed better representation of system variance and clustering of samples based on molecular fingerprinting. Before chemometric procedures, each raw spectrum was mean-normalized [34] and treated with the second derivative with a Savitzky–Golay filter (11 points of smoothing, 2nd order) to reveal the hidden information in the spectra as well as to reduce the noise in the data.

## 3. Results and Discussion

### 3.1. Spectral Analysis of Hop Leaves 

In the present study, for the first time, an in-depth ATR-FTIR analysis of leaves from different hop varieties subjected to different drying treatments was performed in order to evaluate the relationship between the spectral fingerprints and the molecular structures of the samples. 

**Figure 1:**
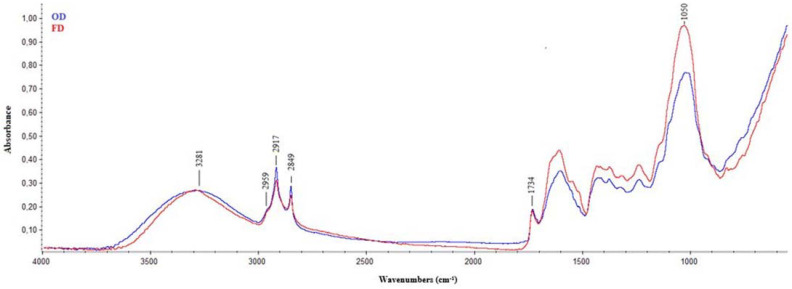
Average IR spectra of samples analyzed. Red line indicates freeze-dried (FD) samples, and blue line indicates oven-dried (OD) ones.

Figure 1 shows the average IR spectra of FD (red line) and OD (blue line) samples, regardless of the genotype analyzed, acquired in the mid-infrared region (wavenumbers from 4000 to 600 cm^−1^), which is the most widely used in the study of biological matrices. This is because each compound has its own unique pattern in the range from 1400 to 600 cm^−1^, often called the fingerprint region, while the region from 1800 to 1400 cm^−1^ provides information about functional groups occurring in the investigated molecules [35]. In this regard, the acquired spectra were analyzed by identifying the positions of the different spectral bands that characterized the samples to identify the signatures of the main functional groups that characterize hop leaves. These are mainly characterized by the presence of fibers (about 32%), proteins (about 8%) and fats (about 4%), which are associated with minor components such as polyphenols, minerals, vitamins, etc. [10,16]. Regardless of the genotype and drying treatment applied, two characteristic regions were clear in all spectra: one above 2800 cm^−1^ and the second below 1800 cm^−1^ (Figure 1). The first broad band, located at about 3281 cm^−1^, was attributed to O–H stretching vibrations occurring in the hydrogen bonds and intermolecular H bonding due to the presence of polysaccharides, non-esterified hydroxyl groups of cutin and phenolic compounds present in hop leaves [15]. Two sharp peaks centered at 2917 and 2849 cm^−1^ were also visible; these peaks were related to the asymmetric and symmetric stretching of -CH_2_ groups, characteristic of lipids and fatty acids in cutin and leaf waxes [36], as well as of cellulose components, interrelated to the lignin molecules [37]. 

A shoulder at about 2959 cm^−1^, attributed to the stretching of −CH_3_ groups, was visible in all spectra acquired. Analyzing the spectral regions indicative of the functional groups and fingerprinting of the sample analyzed, the spectral band centered at 1734 cm^−1^ was attributed to the stretching of the C=O bonds in saturated esters and δ-lactones, which originated from esterified cutin polymer, waxes or polysaccharides [38]. The presence of esters commonly found in the membrane lipid (i.e., cutin) and cell wall pectin [39] was also confirmed by the strong absorption at 1025 cm^−1^ (stretching of COO-C). Finally, in the region between 1100 and 1000 cm^−1^, there were several vibrations of groups, such as C-H bending or C-O or C-C stretching, which are characteristic of cellulose in leaves [40]. 

Regarding the drying treatments, the analysis of average spectra highlighted a difference in the IR signature only in relation to the intensity of the spectral bands, which suggested a detrimental effect of the OD treatment on the overall molecular profile of hop leaves, regardless of the genotype considered, especially in the zone related to cellulose and hemicelluloses, which therefore seemed to undergo a certain degree of degradation as the drying temperature increased, as well as possible changes in the supramolecular organization of cellulose (Figure 1; [41]).

For a better interpretation of the spectral signals below 1700 cm^−1^, derivatization (second derivative) was performed (Figure S1), which allowed the analysis of overlapping peaks present in this spectral region. Derivatization enables the establishment of the exact position of peaks on the IR spectrum, because the minima in the derivative plot correspond to the bands’ maxima [42]. It enriches the spectral features by unveiling a complex of absorption bands and allows differentiation of the samples analyzed according to the drying treatments applied. In both cases (OD: blue line; FD: red line), spectral derivatization confirmed the strong absorption peak centered at 1734 cm^−1^ related to the C=O stretching mode of esters, also revealing the presence of three other medium peaks related to the stretching mode of carbonyl groups interacting through hydrogen bonds at about 1714, 1694 and 1681 cm^−1^ (C=O stretching mode of carboxylic acids interacting through strong hydrogen bonds), all of them attributable to the cutin matrix [36]. 

Two strong peaks centered at 1659 and 1631 cm^−1^ for OD samples and 1651 and 1637 cm^-1^ for FD ones related to amide I (C=O and C-N stretching modes of peptide bond) were also visible (Figure S1; [43]). In addition, a medium-intensity amide II peak (N-H bending and C-N stretching modes of amide bonds) was also visible at 1547 and 1543 cm^−1^ for OD and FD, respectively [42]. Interestingly, OD samples showed a strong peak centered at 1463 cm^−1^, related to the symmetric bending vibration of the -CH_2_ group in fatty compounds, which appeared much less pronounced in FD samples. The influence of drying treatments could be observed in the spectral differences recorded in the carbohydrate spectral region (1500–800 cm^−1^), where a strong reduction in the spectral absorption of OD samples compared to FD ones was observed. A strong peak was observed in all spectra centered at about 1370 cm^−1^ (Figure S1), which was related to the C-H vibrations and CH_2_ bending of cellulose and hemicelluloses [44], which appeared less pronounced in OD samples than in FD ones. Slight differences due to the different thermal treatments applied could also be observed at about 1100 cm^−1^ (C–O stretching, C–C stretching ring pectin) and at 988 cm^−1^ (C–O stretching, C–C stretching cellulose (C6–H2–O6)) [44]. Finally, differences were also observed among treatments at about 835 cm^−1^, which was associated with the vibrations of double bonds and aromatic molecules that could be attributed to the phenolic compounds present in the cuticle of hop leaves [45].

**Figure 2:**
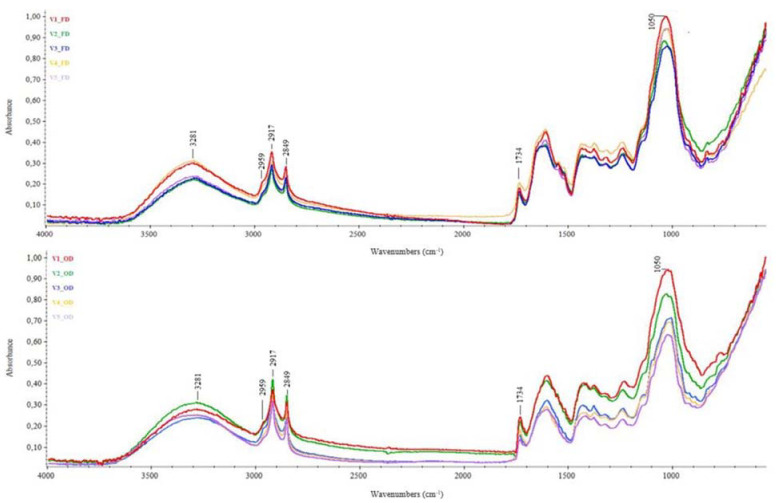
Average IR spectra of *Humulus lupulus* leaves from different varieties: influence of genotype on the applied treatment. FD—freeze-dried samples; OD—oven-dried samples. V1—Chinook hop leaves; V2—Centennial hop leaves; V3—Comet hop leaves; V4—Columbus hop leaves; V5—Cascade hop leaves.

Figure 2 shows the influence of hop varieties on the average IR spectra of OD and FD samples. As can be seen, the overall spectral fingerprint did not seem to be affected by the considered variety, which rather affected the intensity of the spectral absorptions, mainly in the region below 1800 cm^−1^, more markedly for OD samples than for FD ones. Among the varieties tested, Centennial hop leaves (V2; Figure S2b) showed the most pronounced differences in the spectral range above 2500 cm^−1^ in response to the thermal treatment applied compared to the other samples analyzed, showing higher absorptions in OD samples (blue line). In contrast, the main spectral differences for the Comet, Columbus and Cascade samples (V3, V4 and V5, respectively) were below 1800 cm^−1^, with higher absorption in FD samples (red lines; Figure S2c,d). In addition, the Chinook samples (V1) seemed to be unaffected by the different treatments applied. To the best of our knowledge, this is the first time that an in-depth FTIR analysis of the influence of drying treatments and genotypes on hop leaves was carried out, meaning further studies will be necessary to better understand the molecular changes underlying the observed spectral fingerprint.

**Figure 3:**
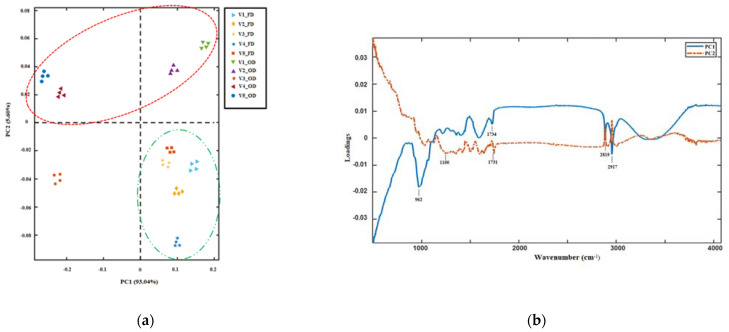
Principal component analysis (PCA) performed on the FTIR spectral dataset in the range 4000–800 cm^−1^: (**a**) score plot of PC1 and PC2; (**b**) loading plot of PC1 and PC2. OD samples are clustered in the red ellipse, while FD ones are in the green ellipse.

The PCA score plot (Figure 3a) and loading plot (Figure 3b) of FTIR spectral data were examined with the aim to discriminate the samples analyzed according to their spectral features. The first two principal components (PCs) explained 98.6% of the total variance. The effect of the drying treatment on the spectral characteristics of hop leaf samples can be clearly visualized in the scoring plot (Figure 3a). OD samples (samples enclosed in the red ellipse) were well separated along PC2 from FD ones (samples enclosed in the green ellipse), with the sole exception of OD Comet leaves (V3). Along PC1 (93.04%), OD samples of Centennial, Comet and Cascade hop leaves (V2, V3 and V5, respectively) were well separated from the other varieties investigated. The PC loading plot indicated that the following wavelengths were responsible for the group separation along PC1: 962, 1734, 2819 and 2917 cm^−1^, all of which were related to the molecular features of cutin, while the spectral region ranging from 1100 to 1731 was responsible for sample grouping along PC2 (Figure 3b), highlighting a structural impact of the different drying treatments applied, mainly involving the cellulose and hemicellulose fractions, on hop leaf samples analyzed.

### 3.2. Phytochemical Screening of Hop Leaves

Hop leaves are reported to be a good source of bioactive compounds that can be further exploited, among other areas, as food supplements and for tea infusions [16]. In this regard, in order to preserve their phytochemicals with respect to their health-promoting properties, the leaves must be dried. To the best of our knowledge, there is no literature data on the effect of drying treatments on hop leaf quality. However, published data on different plant matrices shows varying effects of different drying methods on their nutritional and nutraceutical characteristics, depending on the genotype, time and temperature of the process and on the chemical-physical mechanisms of water removal from the plant matrix [26]. In this study, acquired phytochemical data on hop leaves subjected to different drying treatments were analyzed using ANOVA (Table 2). The results highlighted that both V and T, as well as V × T interactions, significantly influenced all parameters studied; T was the main factor contributing to the total variation in the overall nutraceutical profile of hop leaves (*p* < 0.001), and V was the main factor contributing to the total variation in their pigment content (*p* < 0.001), in line with literature findings [17,46]. 

**Figure 4:**
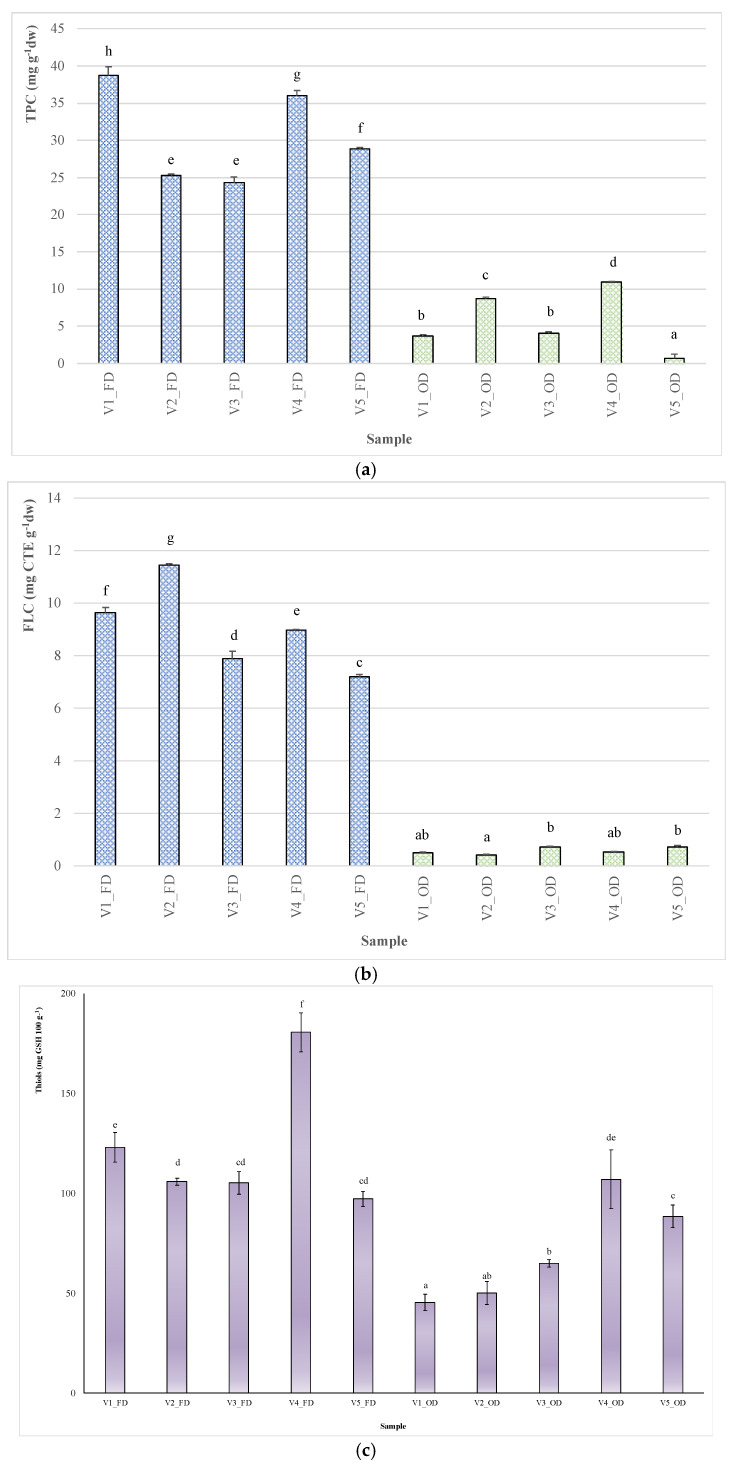

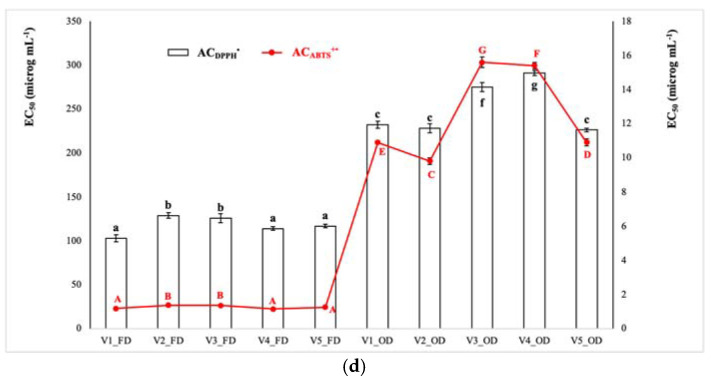
Influence of genotype and drying treatment on the phytochemical composition of samples analyzed: (**a**) total phenol content (TPC); (**b**) total flavan content (FLC); (**c**) total thiol content (Thl); (**d**) total antiradical capacity (AC) evaluated by DPPH^•^ and ABTS^•+^ in vitro assays. OD—oven-dried samples; FD—freeze-dried samples. V1—Chinook hop leaves; V2—Centennial hop leaves; V3—Comet hop leaves; V4—Columbus hop leaves; V5—Cascade hop leaves. Different letters indicate significant differences in the mean (*p* < 0.05). Within each graph different letters indicate significant differences in the mean (*p* < 0.05). In (**d**), lowercase letters indicate significant differences in the mean (*p* < 0.05) among AC_DPPH_^•^ values, while uppercase letters indicate significant differences in the mean (*p* < 0.05) among AC_ABTS_^•+^.

The results for total phenols and flavans are shown in Figure 4 (a and b, respectively). Among the tested samples, TPC ranged from 0.69 ± 0.01 to 39 ± 1 mg GAE g^−1^ (for V5_OD and V1_FD, respectively; Figure 4a). 

Regarding treatments, on average, OD samples showed significantly lower TPC than FD ones (−82%), as also observed in the literature data on different plant matrices [47,48]. Among FD samples, Chinook showed the highest TPC (V1; 39 ± 1 mg GAE g^−1^), followed by Columbus (V4; 35.9 ± 0.8 mg GAE g^−1^) and Cascade (V5; 28.9 ± 0.2 mg GAE g^−1^). A significant influence of the genotype on the TPC of FD hop leaves was also reported by Iglesias et al. [15]. Among OD samples, the highest TPC was recorded for Columbus (V4; 10.9 ± 0.2 mg GAE g^−1^), followed by Centennial (V2; 8.7 ± 0.2 mg GAE g^−1^). On average, these results are higher than those reported by Ceh et al. [49] for leaves from different hop varieties oven-dried at 45 °C.

FD leaves also showed the highest FLC, ranging from 11.46 ± 0.04 (Centennial) to 7.22 ± 0.04 mg CTE g^−1^ (Cascade), while the lowest flavan value was recorded for OD Centennial samples (0.42 ± 0.03 mg CTE g^−1^) (Figure 4b). According to Samoticha et al. [50], low temperature and low oxygen levels, as in the case of freeze-drying, ensure better preservation of bioactive compounds. Free soluble thiols, such as glutathione, cysteine, and homocysteine, are a class of organic sulfur derivatives (mercaptans) with sulfhydryl functional groups (−SH), which play a crucial role in protecting cells from oxidative damage. In fact, biological thiols directly control reactive oxygen species (ROS) production and are involved in ascorbate pool recycling through a sequence of reactions collectively known as the Halliwell–Asada or ascorbate–glutathione cycle [33]. Fruits and vegetables are the largest source of dietary thiols, such as cysteine and glutathione, while animals are unable to assimilate inorganic sulfur and produce cysteine from methionine in the way that plants can. In this sense, thiols determination in hop leaves appears important for their possible use as food additives or in the formulation of functional drinks, such as teas. The results for total thiols are shown in Figure 4c. On average, Thl ranged from 45 to 180 mg 100 g^−1^ for V1_OD and V4_FD, respectively. As expected, significant Thl reduction was observed in OD samples (on average, −40% compared to FD samples). Thermal treatments, in fact, are known to induce rapid antioxidant metabolite degradation, as stated above for polyphenols. A continuous decline in GSH was also observed in apple leaves exposed to 40 °C for more than 4 h, compared to similar leaves grown at a lower temperature [51]. Moreover, our results agree with the study of Paolo et al. [52], where a strong decrease in the levels of glutamic acid (i.e., the amino acid that forms glutathione together with cysteine and glycine) in dried tomato fruits was observed following oven drying (−94%) compared to freeze-drying. Among FD samples, Columbus (V4) showed the highest Thl level (180.5 ± 12.1 mg GSH 100 g^−1^), followed by Chinook (V1; 122.9 ± 9.1 mg GSH 100 g^−1^), while the other three varieties had similar contents of around 100 mg GSH 100 g^−1^. To the best of our knowledge, no study has been published on determining the levels of thiol compounds in hop leaves. However, our data indicate that hop leaves are a good source of thiols, since their levels are similar to those found by Mills et al. [53] in vegetables such as tomato and cauliflower (i.e., around 3 µmol GSH g^−1^ dw).

Pigments are an important class of plant bioactive compounds, showing significant antioxidant potential against hydroperoxide generation [54]. Chlorophyll’s consumption has been associated with protective effects against several degenerative disorders, such as atherosclerosis, osteoporosis, cataracts, neurodegenerative diseases and oxidative stress [55]. In addition, chlorophyll is the main compound that determines the greenness of dry leaves, whose retention could represent an important quality parameter for commodity purposes. In the present study, three classes of hop leaf pigments were analyzed: chlorophyll *a*, chlorophyll *b*, as well as their sum, and total carotenoids (Table 3). As expected, FD samples, on average and regardless of the genotype, were characterized by a total chlorophyll content about 1.5 times higher than OD samples, with greater retention of both Chl *a* and *b* (+ 32% and + 30%, respectively) compared to the same pigments in OD samples. The highest total chlorophyll content was found in FD Comet and Columbus samples (V3 and V4, respectively; Table 3), whose average content (about 1.31 mg g^−1^) is in line with the average content of total chlorophyll of green tea leaves (1.47 mg g^−1^), known for their high antioxidant potential [56]. The authors also reported significant effects of the genotype and growth environment, as well as postharvest handling, on the pigment content of tea leaves. 

Regarding the different types of chlorophylls, the highest value of Chl *a* was found in V4_FD samples (441 ± 3 μg g^−1^), while the lowest was observed in V1_OD samples (104 ± 1 µg g^−1^). Among the varieties analyzed, Centennial samples (V2) showed a negligible, although statistically significant, effect of drying methods on Chl *a* content (360 ± 2 and 352 ± 1 µg g^−1^ for V2_FD and V2_OD, respectively), whereas oven drying exerted a strongly negative effect on the Chl *a* content of Columbus hop leaves (V4), reducing its content by approximately 4-fold compared with FD samples of the same variety. For Chl *b*, the highest value was found in the V3_FD sample (883 ± 5 µg g^−1^), while the lowest one was observed in the V5_FD sample (285 ± 3 µg g^−1^). The influence of the different drying processes on chlorophyll pigments in hop leaves is in line with the literature data on dried herbs [19,21] and highlight a significant effect of the genotype on their susceptibility to thermal degradation (Table 2).

Carotenoids are well-known lipophilic compounds that act as radical scavengers and vitamin A precursors able to prevent various cancer and age-related diseases [57]. The TC content of hop leaves ranged from 91 ± 3 to 266 ± 4 μg g^−1^ for V1_OD and V5_OD, respectively, which, for the latter, was in line with the TC of dried tea (on average, 247 µg g^−1^; [58]). In addition, oven drying had a detrimental effect on the TC content for both Chinook (V1) and Columbus (V4) samples, with a reduction of 52 and 27.5%, respectively, compared with FD ones. To the best of our knowledge, there are no reports in the literature on the TC content of hop leaves.

### 3.3. Antiradical Capacity (AC)

Figure 4d shows the antiradical potential of hop leaf extracts against the synthetic chromogenic radicals DPPH^•^ and ABTS^• +^. The results were expressed indirectly by measuring the quantity of plant extract necessary to reduce the initial synthetic radical concentration by 50%, which is a value defined as EC_50_: the higher the antioxidant potential of the extract, the lower this value is.

On average, AC_DPPH_ ranged from 103 to 291 μg mL^−1^ for V1_FD and V4_OD, respectively, while AC_ABTS_ ranged from 1.15 to 15.6 μg mL^−1^ for V4_FD and V3_OD, respectively. In both in vitro tests, the highest AC values were recorded for FD samples, regardless of the genotype considered. Among genotypes, V1_FD and V4_FD showed the highest AC, while V3_OD and V4_OD showed the lowest one, regardless of the in vitro test used. Simple correlation analysis revealed that the scavenging activity of the extracts analyzed showed high negative correlations with FLC (*r* = −0.923 for AC_ABTS_ vs. FLC, *p* < 0.01; *r* = −0.912, *p* < 0.01, for AC_DPPH_ vs. FLC) and TPC (*r* = −0.901 for AC_DPPH_ vs. TPC, *p* < 0.01; *r* = −0.877, *p* < 0.01, for AC_ABTS_ vs. TPC) and weaker, but highly significant, negative correlations with total thiols (*r* = −0.639 for AC_DPPH_ vs. Thl, *p* < 0.01; *r* = −0.583, *p* < 0.01, for AC_ABTS_ vs. Thl).

### 3.4. Exploratory Data Analysis

**Figure 5:**
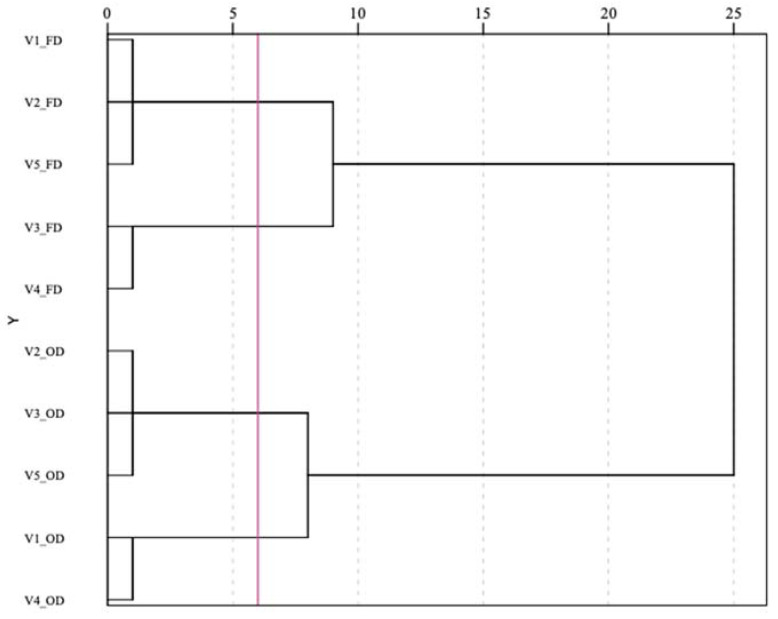
Hierarchical cluster analysis for the *Humulus lupulus* leaf extracts analyzed. OD—oven-dried samples; FD—freeze-dried samples. V1—Chinook hop leaves; V2—Centennial hop leaves; V3—Comet hop leaves; V4—Columbus hop leaves; V5—Cascade hop leaves.

To simplify the chemical pattern recognition and visualize relationships between varieties and treatments, hierarchical cluster analysis (HCA) was used (Figure 5). Cluster analysis is a way of grouping samples based on the similarity of responses to the parameters analyzed; a dendrogram was produced to visualize the clustering process. A general separation was observed between the OD and FD samples at an average distance between 10 and 15 (two main clusters) due to the overall higher scores of the latter for all variables considered. In addition to this, a further division of samples into four subclusters could be observed at an average distance between 5 and 7 (red line; Figure 5), highlighting the existence of subtle differences between samples related to genotype. This further classification showed that the heat treatments studied had different effects depending on the variety considered, emphasizing that freeze-dried Comet and Columbus samples (V3_FD and V4_FD, respectively) were those with the highest chlorophyll content (cluster 2 in Table 4), while oven-dried Chinook and Columbus samples (V1_OD and V4_OD, respectively) were characterized by the worst overall phytochemical profile (cluster 3 in Table 4). Furthermore, Chinook, Centennial and Cascade hop leaves (V1, V2 and V5, respectively), when freeze-dried, showed the best overall phytochemical profiles. Finally, oven-dried Centennial, Comet and Cascade samples (V2_OD, V3_OD and V5_OD, respectively) were characterized by the highest content of total carotenoids.

**Figure 6:**
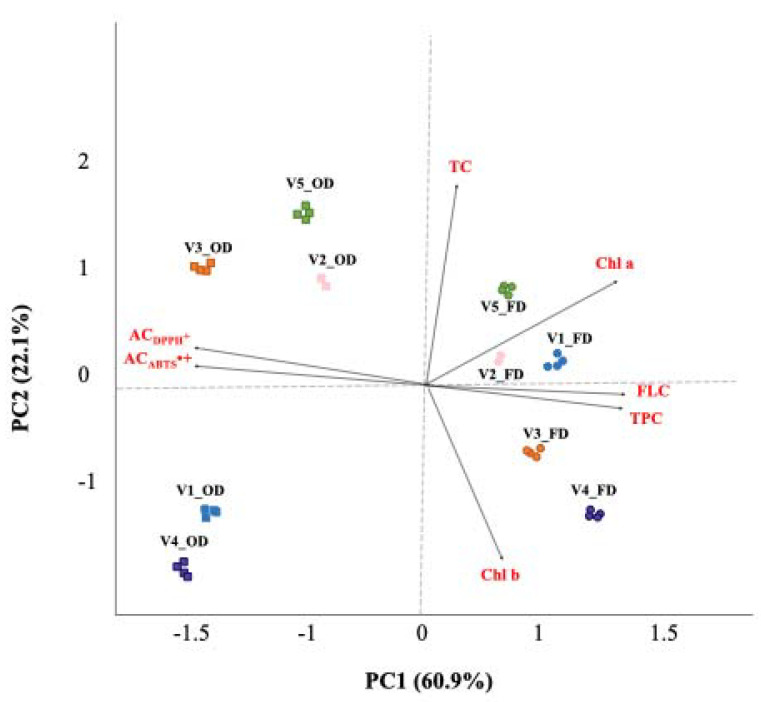
Biplot obtained by combining information from nutraceutical and antioxidant properties of samples analyzed. OD—oven-dried samples; FD—freeze-dried samples. V1—Chinook hop leaves; V2—Centennial hop leaves; V3—Comet hop leaves; V4—Columbus hop leaves; V5—Cascade hop leaves. TPC—total phenolic content; FLC—total flavan content; Chl *a*—chlorophyll *a*; Chl *b*—chlorophyll *b*; Chl tot—total chlorophyll; AC_ABTS•+_—antiradical capacity (ABTS in vitro test); AC_DPPH•_—antiradical capacity (DPPH in vitro test).

Furthermore, PCA was conducted on selected variables (Table 5), chosen based on the analysis of the correlation and anti-image correlation matrices obtained from the standardized z-scores, with orthogonal rotation (varimax model). The Kaiser–Meyer– Olkin measure verified the sampling adequacy for the analysis (KMO = 0.740). Bartlett’s test of sphericity (*p* < 0.001) showed that correlations between the considered items were sufficiently large for PCA. Based on eigenvalues > 1 (Kaiser’s criterion) and a scree plot (not shown), two principal components (PCs), accounting for 83.0% of the total variance, were considered significant. The PCA biplot shown in Figure 6 shows clusters of samples based on their similarity. Samples were grouped according to the different drying treatments along PC1, while PC2 allowed the separation of samples based mainly on their TC content, according to HCA results (Table 4). The PCA biplot, in fact, shows both the PC scores of samples (symbols) and the loadings of variables (vectors). The further away these vectors are from a PC origin, the more influence they have on that PC. Loading plots also hint at how variables correlate with one another: a small angle implies a positive correlation, a large one suggests a negative correlation, and a 90° angle indicates no correlation between the two characteristics. In this analysis, the first component (PC1), which accounted for 60.9% of the total variance, was strongly associated with both total phenols (factor loading: 0.913) and flavans (factor loading: 0.946), which were inversely correlated with the antioxidant potential of samples, while the second component (PC2), which accounted for 22.1% of the total variance, was mainly associated with the carotenoid content of the samples analyzed (factor loading: 0.983). Finally, it is worth noting that the PCA results for phytochemical traits were in line with the PCA results for spectral data, indicating that ATR-FTIR is a valuable green technique to evaluate the phytochemical profiles of plant extracts.

## 4. Conclusions

At present, agricultural production generates large quantities of organic waste as early as the harvesting stage. In the case of hop cultivation, agricultural waste, mainly comprises leaves and stems, accounting for around two-thirds of the harvest. This biomass is currently underexploited and can be therefore revalued as a natural source of ingredients to be used in the food, cosmetics, or pharmaceutical industries, in line with the European Community’s guidelines for increasing sustainable agriculture. However, it must be processed by drying to ensure shelf-stable products. 

The results presented in this study indicate the presence of both soluble polyphenolic compounds and antioxidant pigments, in the non-phenolic fraction of the biomass from hop leaves, in quantities comparable to or, in some cases, higher than the values reported in literature for dried herbs such as green tea. However, further research is needed to evaluate the presence and related contents of high-value human-health-promoting compounds, such as alpha/beta acids and xanthohumol derivatives.

Moreover, a strong interaction between the drying treatment applied and the genotype considered was observed. The overall nutraceutical profile of hop leaves was better preserved when freeze-drying was used, although a higher content of carotenoids was retained in OD samples when Centennial, Comet and Cascade varieties were considered. The choice of the drying method to be adopted, in our opinion, should then be made considering both the possibility of amortizing the equipment already present on the farm and the intended use of the product.

Finally, the present findings reveal, for the first time, the ability of ATR-FTIR spectroscopy to quickly and easily discriminate hop leaf samples according to the treatments applied, and highlight the significant changes they induce in the cellulose, hemicellulose and cutin matrix of hop leaves. The results from this study, although preliminary, open new perspectives on the possible use of hop leaves to recover, through a green approach, natural bioactive compounds to be used as high-value additives in the food sector as well as in other industries, responding to the growing demand for plant-based products instead of synthetic additives. Moreover, drying hop leaves directly on the farm could offer hop producers the possibility to diversify farm income by making herbal teas by taking advantage of the equipment in their possession, thus realizing a circular approach to farm management.

## Figures and Tables

**Table 1:** List of samples analyzed.

Hop Variety	Code
Chinook	V1
Centennial	V2
Comet	V3
Columbus	V4
Cascade	V5

**Table 2:** Factorial analysis based on phytochemical traits of leaf samples from different hop varieties subjected to different drying treatments.

	DF	TPC	FLC	AC_ABTS_	AC_DPPH_	Chl *a*	Chl *b*	Chl tot	TC	Thl
Variety (V)	4	131.6 **	4.6 **	15.5 **	1566.9 **	1806.1 ***	6477.6 ***	80349 ***	4148.5 ***	5751.4 **
Treatment (T)	1	6267.5 ***	715.7 ***	1283.7 ***	162,002 ***	12,301 **	29,401 **	1105 **	470 **	26,191.2 ***
V × T	4	103.2 **	6.3 **	15.9 **	1313.7 **	1871.3 **	6566.9 **	1120 **	456 **	1575.7 **
Error	30	0.35	0.01	0.01	206.4	1.4	4.4	5.2	1.8	50.3

DF—degrees of freedom; TPC—total phenolic content; FLC—total flavan content; AC_ABTS_—antiradical capacity (ABTS in vitro test); AC_DPPH_—antiradical capacity (DPPH in vitro test); Chl *a*—chlorophyll *a*; Chl *b*—chlorophyll *b*; Chl tot—total chlorophyll; TC—total carotenoids; Thl—total thiols. ** *p* < 0.01; *** *p* < 0.001.

**Table 3:** Pigment content (mean ± SD) of *Humulus lupulus* leaf extracts.

Sample	Chlorophyll *a* (μg g^−1^ dw)	Chlorophyll *b* (μg g^−1^ dw)	Total Chlorophyll (μg g^−1^ dw)	Total Carotenoids (μg g^−1^ dw)
V1_FD	320 ± 2c	367 ± 3e	687 ± 5e	191 ± 2e
V2_FD	360 ± 2e	389 ± 1g	749 ± 3g	182 ± 2d
V3_FD	433 ± 1f	883 ± 5l	1316 ± 6i	166 ± 2c
V4_FD	441 ± 3g	857 ± 5i	1298 ± 8h	131 ± 1b
V5_FD	266 ± 2b	285 ± 3a	551 ± 5b	222 ± 1f
V1_OD	104 ± 1a	376 ± 2f	480 ± 3a	91 ± 3a
V2_OD	352 ± 1d	339 ± 2c	692 ± 3e	218 ± 3f
V3_OD	319 ± 1c	316 ± 2b	635 ± 3c	231 ± 1g
V4_OD	108 ± 1a	557 ± 2h	665 ± 3d	95 ± 2a
V5_OD	363 ± 2e	358 ± 6d	721 ± 8f	266 ± 4h

FD—freeze-dried samples; OD—oven-dried samples. a V1—Chinook hop leaves; b V2—Centennial hop leaves; c V3—Comet hop leaves; d V4—Columbus hop leaves; e V5—Cascade hop leaves. Different letters in a column indicate significant differences in the mean (*p* < 0.05).

**Table 4:** ANOVA results of hierarchical cluster analysis (mean ± SD).

Variable	Cluster 1	Cluster 2	Cluster 3	Cluster 4
TPC	30.9 ± 0.7b	30.2 ± 0.2b	7.3 ± 0.4a	4.5 ± 0.4a
FLC	9.4 ± 0.4b	8.4 ± 0.2b	0.52 ± 0.03a	0.62 ± 0.01a
Chl *a*	315.3 ± 0.4b	437.1 ± 0.4c	105.9 ± 0.5a	345.1 ± 0.1b
Chl *b*	347 ± 5a	870 ± 3b	466 ± 2a	337 ± 3a
Chl tot	662 ± 2a	1307 ± 4b	573 ± 3a	683 ± 2a
TC	198 ± 2b	149 ± 1a	93 ± 1a	238 ± 3c
AC_DPPH_	118.1 ± 0.6a	120.6 ± 0.4a	250.5 ± 0.5b	243.6 ± 0.7b
AC_ABTS_	1.3 ± 0.1a	1.2 ± 0.1a	13.2 ± 0.3b	12.2 ± 0.2b

TPC—total phenolic content, data are expressed as mg GAE g^−1^; FLC—total flavan content, data are expressed as mg CTE g^−1^; AC_ABTS_—antiradical capacity (ABTS in vitro test), data are expressed as EC_50_ in µg mL^−1^; AC_DPPH_—antiradical capacity (DPPH in vitro test), data are expressed as EC_50_ in µg mL^−1^; Chl *a*—chlorophyll *a*, data are expressed as µg g^−1^; Chl *b*—chlorophyll *b*, data are expressed as µg g^−1^; Chl tot—total chlorophyll, data are expressed as µg g^−1^; TC—total carotenoids, data are expressed as µg g^−1^. Different letters in a row indicate significant differences in the mean (*p* < 0.05).

**Table 5:** Loadings of the significant measured variables on the two principal components (PCs) *.

Variables	Components	
	1	2
TPC	0.913	
FLC	0.946	
AC_ABTS_	−0.965	
AC_DPPH_	−0.960	
Chl *a*	0.680	0.449
Chl *b*	0.441	−0.582
TC		0.983
Eigenvalues	4.3	1.5
% of variance	60.9	22.1

Extraction method: principal component analysis. * Rotation method: varimax with Kaiser normalization. Component loadings with absolute values less than 0.4 have been left out of the table for ease of comparison. TPC—total phenolic content; FLC—total flavan content; AC_ABTS_—antiradical capacity (ABTS in vitro test); AC_DPPH_—antiradical capacity (DPPH in vitro test); Chl *a*—chlorophyll *a*; Chl *b*—chlorophyll *b*; TC—total carotenoid content.

## Data Availability

Data is contained within the article or supplementary material.

## Supplementary Materials

The following are available online at https://www.mdpi.com/article/10.3390/plants11010099/s1, Figure S1: Second derivative IR spectra of samples analysed. Red line indicates freeze-dried (FD) samples and blue line oven-dried (OD) ones. Figure S2. Influence of drying treatments on the IR fingerprinting of samples analysed. Red line indicates freeze-dried (FD) samples and blue line oven-dried (OD) ones.
