# Supplementary material for: Hop Leaves as an Alternative Source of Health-Active Compounds: Effect of Genotype and Drying Conditions
Source: Plants (Basel). 2021 Dec 29;11(1):99. doi: 10.3390/plants11010099 (PMC8747731; doi:10.3390/plants11010099)

Figure S1. Second derivative IR spectra of samples analyzed. Red line indicates freeze-dried (FD) samples and blue line oven-dried (OD) ones.

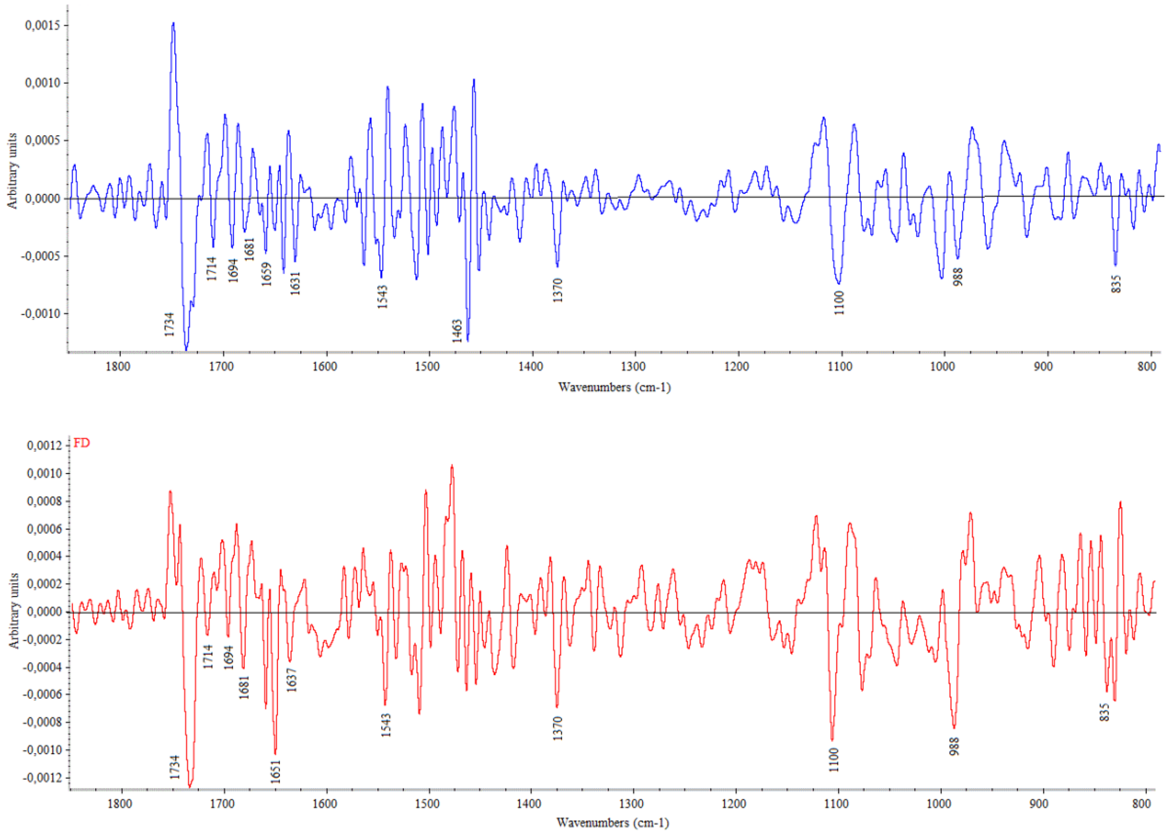

Supplement: Supplementary file 1 [file plants-11-00099-s001.zip › Figure S1.pdf]
