# Supplementary material for: Hop Leaves as an Alternative Source of Health-Active Compounds: Effect of Genotype and Drying Conditions
Source: Plants (Basel). 2021 Dec 29;11(1):99. doi: 10.3390/plants11010099 (PMC8747731; doi:10.3390/plants11010099)

Figure S2. Average IR spectra of *Humulus lupulus* leaves from different varieties: influence of drying treatments on each hop variety analysed. FD: freeze-dried samples; OD: oven-dried samples. A) V1: Chinook hop leaves; b) V2: Centennial hop leaves; c) V3: Comet hop leaves; d) V4: Columbus hop leaves; e) V5: Cascade hop leaves.

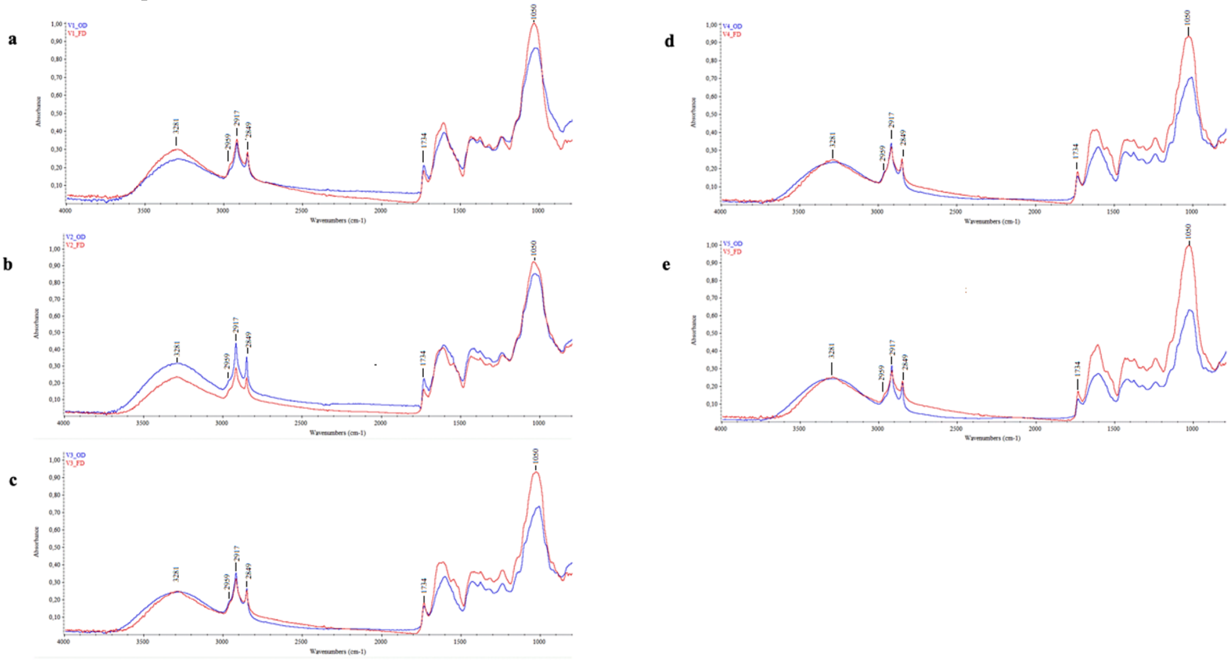

Supplement: Supplementary file 1 [file plants-11-00099-s001.zip › Figure S2.pdf]
